# CCR5 Δ32 and CTLA-4 +49 A/G Gene Polymorphisms and Interferon-β Treatment Response in Croatian and Slovenian Multiple Sclerosis Patients

**DOI:** 10.3390/ijms25137412

**Published:** 2024-07-05

**Authors:** Jasna Nekić, Ivana Stanković Matić, Valentino Rački, Dolores Janko Labinac, Vladimira Vuletić, Miljenko Kapović, Smiljana Ristić, Borut Peterlin, Nada Starčević Čizmarević

**Affiliations:** 1 Department of Nuclear Medicine, Clinical Hospital Center Rijeka, 51000 Rijeka, Croatia; jasna.nekic@medri.uniri.hr; 2 Department of Medical Biology and Genetics, Faculty of Medicine, University of Rijeka, 51000 Rijeka, Croatia; ivanasm@uniri.hr (I.S.M.); miljenko.kapovic@uniri.hr (M.K.); smiljana.ristic@uniri.hr (S.R.); 3 Department of Neurology, Faculty of Medicine, University of Rijeka, 51000 Rijeka, Croatia; valentino.racki@medri.uniri.hr (V.R.); vladimira.vuletic@medri.uniri.hr (V.V.); 4 Department of Neurology, Clinical Hospital Center Rijeka, 51000 Rijeka, Croatia; 5 Department of Neurology, General Hospital Center Pula, 52100 Pula, Croatia; dolores_janko@net.hr; 6 Clinical Institute of Genomic Medicine, University Medical Centre Ljubljana, 1000 Ljubljana, Slovenia; borut.peterlin@guest.arnes.si

**Keywords:** multiple sclerosis, CCR5 Δ32, CTLA-4 +49 A/G, polymorphism, IFN-β, treatment response

## Abstract

The aim of the present study was to investigate the impact of CCR5 Δ32 and CTLA-4 polymorphisms on the response to IFN-β treatment in our cohort of MS patients from Croatia and Slovenia. Genomic DNA was obtained from 295 MS patients (230 female; 65 male) classified as responders (*n* = 173) and non-responders (*n* = 122) based on clinical criteria for treatment efficacy. Genotyping was performed via PCR/PCR-RFLP. No significant differences in the genotype/allele frequencies of CCR5Δ32 and CTLA-4 +49 A/G were detected between male responders and non-responders. A significantly higher prevalence (*p* = 0.039) of the CTLA-4 +49 AA genotype was found in female responders (42.1%) compared to non-responders (28.9%). Using multiple forward regression analysis, the CTLA-4 +49 AA genotype significantly predicted a positive response to IFN-β therapy in females (*p* = 0.011) and contributed to 4.5% of response variability. Furthermore, the combined presence of the CCR5Δ32 wtwt/CTLA-4 +49 AA genotype significantly predicted a positive response to treatment in females (*p* = 0.025). The age at disease onset, pretreatment relapse rate, and baseline EDSS score were not reliable predictors of treatment response in MS patients. Our results indicate that the presence of the CCR5Δ32 polymorphism was not associated with the response to IFN-β treatment, whereas the CTLA-4 +49 polymorphism showed a positive correlation with an optimal response in female patients.

## 1. Introduction

Multiple sclerosis (MS) is an autoimmune and neurodegenerative disease triggered by environmental factors in genetically predisposed individuals. Disease onset is usually between the ages of 20 and 50 years with a female predominance (female:male ratio 2–3:1) [1]. The era of disease-modifying treatments (DMTs) for MS began with the regulatory approval of interferon beta-1b (IFN-β-1b) and interferon beta-1a (IFN-β-1a), representing pioneering therapies for the management of relapsing forms of the disease. Notably, subcutaneous administration of IFN-β-1a, marketed as Rebif^®^ (Rockland, MA, USA), emerged as the third IFN therapy, receiving approval in 2002 [2]. Pegylated formulations of IFN-β-1a, available in both subcutaneous and intramuscular versions under the brand name Plegridy^®^ (Cambridge, MA, USA), were subsequently sanctioned in 2014 and 2021, respectively [3]. These formulations, characterised by extended half-lives, necessitate reduced dosing frequencies, enhancing patient compliance and convenience. Despite the multitude of new drugs available for the treatment of MS, IFN-β still occupies a prominent position [4] due to its combined immunomodulatory, anti-inflammatory, and anti-proliferative properties. Clinical studies have consistently demonstrated the efficacy of IFN-β in reducing clinical and radiological disease activity and slowing disability progression in MS patients. However, 30–50% of patients do not respond to IFN-β treatment [5].

The response to IFN-β therapy is a complex polygenic mechanism that is still poorly understood. Considerable efforts have been made in pharmacogenomic studies focusing on candidate genes, with several genome-wide association studies (GWASs) aimed at identifying allelic variants that could potentially influence the response to IFN-β. The selection of candidate genes in these studies was based predominantly on the hypothesised mechanisms of action of IFN-β and/or significant findings from the GWASs. Some of the candidate genes that have been investigated are HLA class II genes; Myxovirus Resistance Protein 1 (MX1); genes encoding interferon receptors, such as Interferon Alpha and Beta Receptor Subunit 1 (IFNAR1), Interferon Alpha and Beta Receptor Subunit 2 (IFNAR2), Interferon regulatory factor 5 (IRF5), and other interferon-stimulated response elements (ISREs); the interferon gamma (IFN-γ) gene; genes encoding chemokine receptors, such as C-C chemokine receptor type 5 (CCR5); genes related to type I IFN and the Toll-like receptors (TLRs) signalling pathway; genes encoding Gamma-Aminobutyric Acid (GABA) and glutamate receptors; genes encoding cytokines and their receptors, tumour necrosis factor receptor superfamily 10A (TNFRSF10A); innate pattern recognition receptors, antigens CD46 and CD58, cytotoxic T lymphocyte antigen 4 (CTLA4), and Hyaluronan and Proteoglycan Link Protein 1 (HAPLN1); Angiotensin-converting enzyme (ACE); and the Apolipoprotein E (APOE) gene [5,6,7,8]. 

A limited number of studies have investigated the same polymorphisms, often with inconsistent results. Therefore, genetic biomarkers reliably associated with the treatment response in MS patients are lacking.

CCR5 and CTLA4 are both key regulators of the immune response and, although they act in different ways and at different stages of the immune response, play an important role in autoimmune diseases, such as MS. Increasing evidence suggests that chemokines and chemokine receptors are involved in the regulation of inflammation by inducing T-cell infiltration and migration across the blood–brain barrier (BBB), which is considered one of the key processes in the pathogenesis of MS. The expression of CCR5 on T cells, monocytes, macrophages, and dendritic cells mediates the transport of immune cells to the inflamed sites of the central nervous system. When activated by RANTES, macrophage inflammatory protein-1 (MIP-1), or magnocellular nucleus of posterior commissure (MCPC), CCR5 induces the expression of inflammatory cytokines [9].

Several studies have shown that CCR5 expression is significantly increased in active demyelinating lesions and in the cerebrospinal fluid of MS patients during relapse [9,10]. The CCR5 gene is located on chromosome 3p21.3, near an MS linkage region. The CCR5 Δ32 allele, which results from a deletion of 32 base pairs in the coding region of the CCR5 gene, leads to the production of a non-functional truncated protein. Patients who are homozygous for the CCR5 Δ32 allele or heterozygous carriers have absent or reduced expression of the CCR5 cell surface protein, leading to reduced migration of leukocytes to lesion sites and downregulated inflammation in the brain [11].

Great interest has also been shown in CTLA-4, an important immunoregulatory molecule that is mainly expressed on the surface of T cells and is crucial for the inhibition of T-cell activation and peripheral tolerance [12]. The CTLA4 gene consists of four exons on chromosome 2q33 and encodes 233 amino acids. The +49 A/G transition causes a change from threonine to alanine at amino acid 17 in the leader peptide and impairs the inhibitory function of CTLA-4. The G allele is associated with lower expression and the A allele with higher expression of CTLA-4 on the surface of T cells [13]. Although meta-analyses of published studies have found no association between the CCR5 Δ32 and CTLA-4 +49 A/G polymorphisms and susceptibility to MS in Europeans [14,15] their influence on clinical expression or the response to treatment cannot be ruled out, especially if they are combined or considered together. For example, some studies have shown that the CCR5 Δ32 mutation is associated with older age at disease onset [16], delayed clinical progression of MS [17,18], and earlier mortality [19], whereas the CTLA-4 +49 A/G polymorphism affects disease course or progression in some populations [13,20,21,22]. Only a few studies have investigated the relationship between the efficacy of IFN-β treatment and polymorphisms in the CCR5 and CTLA4 genes. The presence of the CCR5 Δ32 allele was associated with an optimal response to IFN-β treatment in Egyptian patients [23], but it had no effect on relapse risk in Danish patients [24]. In Russian patients, polymorphic variants of immune response genes, such as those encoding CCR5, IFNAR1, TGFB1, DRB1, and CTLA-4, were investigated for their effects on the efficacy of IFN-β treatment [25]. The presence of the CCR5 Δ32 allele alone or in combination with specific alleles of other genes (such as CCR5 ∗ Δ32 + IFNAR1 ∗ G + IFNB1 ∗ T/T or CCR5 ∗ Δ32 + IFNAR1 ∗ G + IFNG ∗ T) was associated with improved efficacy of IFN-β treatment. Although no significant association was observed between the single CTLA4+49 A/G allele/genotype and response to treatment, an interesting interaction was noted. Specifically, co-carriage of the CCR5 wt/wt genotype with the CTLA-4 +49G allele enhanced the significance of the association with a non-response to IFN-β treatment (*p* = 0.0028) 12.9-fold compared to the presence of a single unfavourable CCR5 wt/wt genotype. 

Due to the limited number of replication studies and the often-conflicting results across different cohorts and regions, the identification of reliable genetic biomarkers for treatment efficacy in MS remains challenging. Our previous research indicated that the CCR5 Δ32 and CTLA-4 +49 polymorphisms do not influence MS susceptibility or its clinical manifestations in Croatian and Slovenian patients [26,27]. However, the potential impact of these polymorphisms on DMT efficacy has not been explored. Therefore, the objective of the present study was to assess the influence of CCR5 Δ32 and CTLA-4 polymorphisms on the response to IFN-β treatment in our patient cohort.

## 2. Results

Of the 295 MS patients enrolled in this study, 173 (58.6%) were classified as responders and 122 (41.4%) as non-responders to IFN-β. The clinical characteristics of the patients are summarised in Table 1. There was an observable trend towards later onset of disease and lower relapse rate in the responders than the non-responders (*p* = 0.083 and *p* = 0.051, respectively). The mean EDSS score at baseline did not differ significantly between the responder and non-responder groups (*p* = 0.623), but non-responders had more severe MS at the study endpoint (EDSS = 3.9 ± 1.6) than responders (EDSS = 2.5 ± 1.6; *p* = 0.0001). When stratified by sex, these trends observed in the overall sample were primarily evident in female patients, but no significant difference in clinical characteristics were observed between male responders and male non-responders.

Table 2 presents the frequencies of the CCR5 Δ32 and CTLA-4 +49 A/G genotypes and alleles with different genetic models (codominant, dominant, recessive, and overdominant) in responders and non-responders. The genotype distribution did not deviate from the Hardy–Weinberg equilibrium in any group. There were no significant differences in genotype distributions or allele frequencies between responders and non-responders in the entire patient cohort or among males. However, a significantly higher prevalence of the CTLA-4 +49 AA genotype was observed in female responders (42.1%) compared to female non-responders (28.9%; *p* = 0.039; OR = 1.79; 95% CI 1.02–3.13). 

A multiple forward regression analysis showed that the presence of the CTLA-4 +49 AA genotype significantly predicted a positive response to IFN-β therapy in female patients (β = 0.211; multiple R2 change: 0.045; *p* = 0.011), contributing to 4.5% of response variability. The significant impact of the AA genotype on the response to IFN-β therapy in this group was confirmed by a partial coefficient of correlation analysis (r = 0.231; *p* = 0.006). In addition, the combined presence of the CCR5 wtwt/CTLA-4 +49 AA genotype significantly predicted a positive response to treatment in female patients (β = 0.188; multiple R2 change: 0.035; *p* = 0.025; partial coefficients r = 0.204, *p* = 0.016).

In contrast, no significant association was observed between the response to IFN-β therapy and a CCR5 Δ32 or CTLA-4 +49 A/G genotype in male patients. In addition, age at disease onset, pretreatment relapse rate, and baseline EDSS score were not associated with treatment response, neither individually nor in combination with the analysed polymorphisms, in males or females.

## 3. Discussion

Treatment with IFN-β is an efficient and safe way of treating MS within the escalating therapy approach, but the broad spectrum of patient responses suggests a nuanced interplay between treatment efficacy and individual genetic predisposition. Therefore, the identification of specific genetic variants and their interactions is critical to understanding the heterogeneous response to IFN-β. In the present study, we analysed the association between the CCR5 Δ32 and CTLA-4 +49 A/G gene polymorphisms and the clinical response to IFN-β treatment in Croatian and Slovenian MS patients.

As CCR5 is prominently expressed on specific immune cell subsets, notably T cells and macrophages, and serves as a crucial mediator in inflammation and immune modulation, an intricate interplay exists between CCR5 and IFN-β in the modulation of inflammatory pathways. This interaction is particularly significant within the context of MS, in which the regulation of inflammatory processes is critical to disease progression and therapeutic intervention. However, outcomes have yielded conflicting results, with certain investigations suggesting a possible association between specific CCR5 genotypes and treatment responsiveness, whereas others have failed to establish a significant correlation [28]. This discrepancy could be due to different study designs, different response criteria in the different research cohorts, different sample sizes, and the ethnic diversity of patients. Intriguingly, the CCR5 Δ32 allele varies geographically, with a clear north–south gradient within the European population, and is most prevalent in Caucasian populations [28]. Despite the recognised impact of CCR5 alleles on the expression of functional surface proteins and their crucial role in leukocyte chemoattraction, the influence of genotype on RNA expression in MS has not been investigated extensively [29].

CTLA-4 serves as a key negative immunoregulatory protein essential for the function of regulatory T cells (Tregs). Tregs play a pivotal role in dampening T-cell activation and proliferation, thereby maintaining immune homeostasis. Heterozygous mutations in CTLA-4 have been associated with a spectrum of clinical manifestations, including autoimmune disorders targeting specific organs, hypogammaglobulinemia, recurrent infections, and susceptibility to certain cancers [30]. In studies comparing healthy controls and patients with relapsing–remitting multiple sclerosis (RRMS) and secondary progressive multiple sclerosis (SPMS) who had not undergone treatment, both RRMS and SPMS patients exhibited a significantly higher proportion of CTLA-4+ CD4+ T cells compared to the healthy control group. This disparity was particularly pronounced in RRMS patients. Moreover, when CD4+ T cells from SPMS patients, and even RRMS patients, were activated with anti-CD3+ rIL-2, they demonstrated an inability to exhibit normal surface CTLA-4 expression [31]. Similarly, when myelin basic protein (MBP)-reactive T cells from MS patients and healthy controls were subjected to stimulation, inhibition of CTLA-4 resulted in a proliferative response and increased cytokine production. However, this effect was considerably attenuated in MS patients, suggesting a disruption of the regulatory function of CTLA-4 in this population [32]. An investigation of memory CD8+ T cells from the cerebrospinal fluid (CSF) of PPMS and RRMS patients revealed age-related differences in CTLA-4 expression. Healthy controls showed an age-associated decline in CTLA-4 expression, whereas MS patients exhibited no CTLA-4 expression, especially in younger individuals with PPMS. This suggests premature immunosenescence in the CD8+ T cells of younger MS patients, with potential clinical and therapeutic implications [33]. Inhibition of CTLA-4 ligands yielded distinct outcomes due to the interplay between B7-1/B7-2 and their receptors, CD28/CTLA-4. This interaction directs precursor cells toward Th1 or Th2 lineages, influencing clinical outcomes. Anti-B7-1 immunoglobulin (Ig) promoted Th2 differentiation in naïve MBP-specific Th precursor cells and mitigated experimental autoimmune encephalomyelitis (EAE). Anti-B7-2 Ig promoted Th1 differentiation, exacerbating EAE both clinically and histologically [34]. Hallal-Longo et al. found that MS patients undergoing IFN-β therapy had increased intracellular CTLA-4 levels in PBMCs, which correlated with reduced proliferation in response to MBP and myelin, and higher lymphocyte apoptosis [35]. Sellebjerg et al. suggested that IFN-β treatment may increase the frequency of CD25-high CD4+ T cells expressing CTLA-4 [36]. Espejo et al. observed that in RRMS patients undergoing IFN-β treatment, there were no changes in T-lymphocyte proliferative response via the CD28/CTLA-4 pathway within the first 3 months. However, following this period, IL-10 production increased, inhibiting the CD80:CD28/CTLA-4 pathway and reducing IL-2 synthesis. This disruption impacts lymphocyte expansion and autoimmunity initiation, highlighting important immunological dynamics in IFN-β therapy for RRMS patients [37]. In a comparative analysis conducted by Sellebjerg et al., they reported a notable contrast in the proportion of CD4+CD25-high T cells expressing CTLA-4 in untreated MS patients compared with healthy controls [36]. Regardless of the IFN-β therapy status, MS patients had a higher percentage of CD4+CD25-high T cells expressing total CTLA-4 (both intracellular and surface expression) than controls. However, untreated MS patients had a greater percentage of CD25-high CD4+ T cells expressing surface CTLA-4 than healthy controls. Furthermore, this percentage increased following IFN-β treatment in MS patients [36].

In our previous investigations involving Croatian and Slovenian patients, we did not detect any discernible influence of the CCR5 Δ32 or CTLA-4 +49 A/G polymorphisms on susceptibility to MS or its clinical phenotypes. Therefore, we concentrated on assessing the impact of these polymorphisms on the response to IFN-β treatment. There is a major challenge in comparing our results with those of other studies, but we focused on comparing the results with Russian patients due to the similar sample sizes, response criteria, and Slavic ancestry. Interestingly, in contrast to the results observed in Russian patients, no discernible influence of the CCR5 Δ32 variant on treatment response was observed in our study. This incongruence could be due to the significantly lower frequency of this variant in our patient cohort, as well as a significantly higher proportion of non-responders in the Russian cohort than in our patients (65% vs. 41%) [25]. Examination of the influence of the CTLA4 +49 polymorphism on the response to IFN-β treatment showed no significant correlations in our overall patient sample or in the Russian cohort. However, in our cohort, the CTLA4 +49 AA genotype significantly predicted a positive response to treatment in female patients, either alone or in combination with the CCR5 Δ32 wtwt genotype. This is in agreement with the A allele and AA genotype being associated with higher expression of CTLA-4 on the surface of T cells. Stratification by sex was not available for the Russian cohort [25]. Intriguingly, age at disease onset, pretreatment relapse rate, and baseline EDSS score did not emerge as reliable predictors of the response to IFN-β treatment, either in isolation or in combination with the analysed polymorphisms, across both male and female patients. Sex disparities manifest in both the clinical presentation and susceptibility to MS, with female patients exhibiting a higher propensity for disease incidence, whereas a greater proportion of male patients tend to develop the primary progressive subtype of MS [38]. Although immunological factors are implicated in these variations, the precise underlying mechanisms remain elusive.

Studies have revealed that Tregs, Th9 cells, and Th17 cells constitute pivotal CD4 T-cell subsets in human autoimmune disorders, including MS and rheumatoid arthritis [39,40]. This underscores the significance of sexual dimorphism in the regulation of the Th cell network. Targets and biomarkers utilised to evaluate sex-specific responses to IFN-β therapy in MS encompass pathways that serve as counterparts in male and female regulatory mechanisms governing immunological and neurological homeostasis, such as the IL-6 and IFN-γ pathways. Further investigation of the molecular mechanisms underlying these associations may offer valuable insights into the development of targeted therapeutic approaches for MS [41]. Significant differences in MS susceptibility and disease progression have been observed between sexes. It is imperative for preclinical research to consider these clinical observations to facilitate the identification and development of novel and more efficacious DMTs. This approach, termed “bedside-to-bench-to-bedside,” underscores the importance of translating clinical insights into laboratory investigations and subsequently translating research findings back into clinical applications to benefit patients [40]. Moreover, given the variations in treatment effectiveness, optimal dosage requirements, and adverse effects of medications between males and females, sex-based considerations are crucial when stratifying patients for therapeutic interventions. Accounting for sex-specific factors in treatment strategies can optimise patient outcomes and ensure tailored and effective management of MS [41].

The present study has some limitations. First, the number of participants, particularly male participants, is relatively small, which limits our conclusions about the effects of the studied polymorphisms on the response to IFN-β treatment. Second, like most pharmacogenetic studies, our study is based on a short observation period (2 years), but we were not able to follow patients for a longer period of time with the same drug, which would certainly have allowed better assessment of the response to IFN-β [42]. On the other hand, the strength of our study lies in its contribution to the existing body of knowledge regarding sex differences in the response to DMTs for MS. By elucidating these differences, our research underscores the importance of considering sex as a variable in the efficacy of MS treatments, not only enhancing our understanding of the nuanced ways in which male and female patients respond to DMTs, but also encouraging further investigation into the underlying mechanisms driving these differences. This, in turn, may inform more personalised and effective treatment strategies for MS in the future and encourage further research on this topic.

## 4. Materials and Methods

A total of 295 patients treated with IFN-β (230 females, 65 males) who fulfilled the revised McDonald’s criteria for MS [43] were recruited by collaborating clinical and genetic centres in Croatia and Slovenia. The clinical criteria for the response to IFN-β were applied after a 2-year treatment period. Patients with relapse onset MS were divided into two groups: responders with no relapses and no progression of the Expanded Disability Status Scale (EDSS) score and non-responders with ≥1 relapse and an increase in the EDSS score of at least 1 point confirmed at the 6-month follow-up [44]. The investigations were carried out following the rules of the Declaration of Helsinki of 1975 (https://www.wma.net/what-we-do/medical-ethics/declaration-of-helsinki/, accessed on 12 June 2024), as revised in 2013. This study received approval from the ethical committees of both centres, and all participants provided written informed consent.

Genotyping of the CCR5 Δ32 and CTLA-4 +49 A/G gene polymorphisms was performed according to the modified polymerase chain reaction (PCR) or PCR-restriction fragment length polymorphism (RFLP) methods as described previously [29,45]. Genomic DNA was extracted from whole blood using a FlexiGene DNA kit 250 (Qiagen GmbH, Hilden, Germany) according to the manufacturer’s instructions. Genotyping of the CCR5 Δ32 polymorphism was performed using the PCR method with the following primers manufactured by Metabion international AG (Planegg, Germany) flanking the region containing 32-bp deletion: forward primer: 5′-CAA AAA GAA GGT CTT CAT TAC ACC-3′ and reverse primer: 5′-CCT GTG CCT CTT CTT CTC ATT TCG-3′ (product length: 189 bp in the wild-type allele and 157 bp in the Δ32 allele). The PCR reaction mixture contained 0.50 μL forward and reverse primers, 10 pmol/μL; 10 mM dNTP (Metabion international AG, Planegg, Germany), final concentration 0.05 mM; 10× PCR buffer, 1.0 μL; 25 Mm MgCl_2_, final concentration 1 mM; Taq polymerase (Applied Biosystems, Waltham, MA, USA), final concentration 1.5 U; deionised water 7.1 μL; and sample DNA with the concentration of 100 μg/mL–0.5 μL. PCRs were performed using the thermocycler Mastercycler (Eppendorf, Hamburg, Germany), and the PCR cycling conditions were as follows: 40 cycles, with five cycles of 94 °C for 1 min, 55 °C for 1 min, and 72 °C for 1.5 min, followed by 35 cycles of 94 °C for 30 s, 60 °C for 30 s, and 72 °C for 45 s. During the electrophoresis in Tris-Acetate plus EDTA buffer (80 V/40 min), PCR products (10 μL) were separated on 3.0% agarose gels stained with ethidium bromide and were visualized under UV at a wavelength of 312 nm. 

Genotype of the CTLA-4 +49 A/G polymorphism was determined by PCR-RFLP using BstE II enzyme (New England BioLabs, Ipswich, MA, USA). The +49 A/G polymorphism was amplified with forward primer 5′-AAGGCTCAGCTGAACCTGGT-3′ and reverse primer 5′-CTGCTGAAACAAATGAAACCC-3′ (Metabion international AG, Planegg, Germany) resulting in a product of 152 bp. The PCR reaction mixture contained 10× PCR buffer, 1.5 μL; 25 Mm MgCl_2_, final concentration 1 mM (Applied Biosystems, Waltham, MA, USA); 10 mM dNTP final concentration 0.05 mM (Metabion international AG, Planegg, Germany); primers, each at final concentration 0.2 μM; Taq polymerase (Applied Biosystems, Waltham, MA, USA), final concentration 1.0 U; deionised water 10.6 μL; and DNA sample at a concentration of 100 μg/mL–0.5 μL. The total reaction volume was 15 μL, and the protocol for PCR amplification was as follows: an initial denaturation step at 94 °C for 5 min, followed by 35 cycles of denaturation at 94 °C for 40 s, annealing at 58 °C for 40 s, extension at 72 °C for 40 s, and final extension at 72 °C for 3 min. The PCR products were digested at 37 °C and analysed by running them in Tris-Acetate plus EDTA buffer (80 V/60 min) on 3% agarose gel stained with ethidium bromide. Restriction fragments were visualized under UV at a wavelength of 312 nm, with the +49 A/G polymorphism determined by a 152 bp fragment (representing the A allele) or two fragments of 132 and 20 bp (representing the G allele).

Statistical analyses were carried out using Statistica for Windows, version 13.3 (StatSoft, Inc., Tulsa, OK, USA). To analyse the association between treatment response and polymorphisms using different genetic models (codominant, dominant, recessive, and overdominant), we employed Fisher’s exact test and Chi-squared test to compare genotype and allele frequencies between groups. The genetic models were defined as follows: codominant (MM vs. Mm vs. mm), dominant (MM vs. Mm + mm), recessive (mm vs. Mm + MM), and overdominant (MM + mm vs. Mm), where M represents the major allele and m represents the minor allele. A multiple forward stepwise regression analysis, including polymorphisms, age at disease onset, relapse rates, and EDSS score prior to IFN-β treatment, was performed to evaluate the independent and combined effects of the analysed polymorphisms on the IFN-β treatment response. Partial coefficients of correlation were calculated to test the correlation between the CCR5 Δ32 and CTLA-4 +49 A/G genotypes and treatment response, controlling for age at onset, relapse rate, and EDSS before IFN-β treatment.

Odds ratios (ORs) and 95% confidence intervals (CIs) were calculated using MedCalc for Windows, version 12.7.7 (MedCalc Software, Mariakerke, Belgium). The Hardy–Weinberg equilibrium was assessed using the Simple Hardy–Weinberg Calculator-Court Lab (Washington State University of Veterinary Medicine, Pullman, WA, USA).

## 5. Conclusions

The principal finding of our study is that the CTLA4 +49 AA genotype is a predictor of a favourable response to IFN-β treatment in female patients. This observation implies that certain genotypes can serve as reliable indicators of treatment efficacy, highlighting the potential role of genetic testing in patient stratification for personalized therapy. This stratification could help identify individuals who are more likely to benefit from IFN-β therapy versus those who may not respond as well. Clinicians could use genetic testing results alongside clinical factors to make more informed decisions about treatment strategies for MS patients; for example, female patients with the CTLA4 +49 AA genotype might be prioritized for IFN-β therapy. Understanding genetic influences could also guide the development of combination therapies that target multiple pathways implicated in MS, potentially enhancing efficacy and reducing side effects.

Our finding also highlights the importance of sex-specific analyses and the need for larger studies with diverse patient populations to validate these findings across different ethnicities and geographical regions. This approach has the potential to improve treatment outcomes by tailoring therapies to individual genetic profiles, ultimately enhancing patient care in the management of MS and potentially other complex diseases.

## Figures and Tables

**Table 1:** Clinical characteristics of the MS patients according to sex and response to IFN-β treatment.

	Males (*n* = 65)			Females (*n* = 230)			Total (*n* = 295)		
Clinical Data	Responders (*n* = 40)	Non- Responders (*n* = 25)	*p*	Responders (*n* = 133)	Non- Responders (*n* = 97)	*p*	Responders (*n* = 173)	Non- Responders (*n* = 122)	*p*
Age at onset, years *	29.2 ± 6.9	28.4 ± 7.6	0.662	28.8 ± 7.8	26.9 ± 8.3	0.098	28.9 ± 7.6	27.2 ± 8.2	0.083
No. of relapses in previous 2 years *	1.6 ± 1.0 (1–5)	1.8 ± 1.1 (1–4)	0.559	1.8 ± 1.2 (1–7)	2.1 ± 1.2 (1–6)	0.074	1.7 ± 1.1 (1–7)	2.0 ± 1.2 (1–6)	0.051
EDSS at baseline *	2.7 ± 1.6 (1–6.5)	2.1 ± 2.2 (1–7.5)	0.355	2.8 ± 1.6 (0.5–7)	3.0 ± 1.3 (1–5.5)	0.349	2.8 ± 1.6 (0.5–7)	2.9 ± 1.5 (1–7.5)	0.623
EDSS at study endpoint *	2.0 ± 1.0 (1–4)	3.1 ± 2.8 (1–7.5)	0.156	2.7 ± 1.7 (0.5–6.5)	4.0 ± 1.5 (1–7)	0.0001	2.5 ± 1.6 (1–6.5)	3.9 ± 1.7 (1–7.5)	0.0001

* Values are given as mean ± SD (range). EDSS, Expanded Disability Status Scale.

**Table 2:** Genotype and allele frequencies of CCR5 Δ32 and CTLA-4 +49 A/G polymorphisms among the MS patients according to sex and response to IFN-β treatment.

	Males (*n* = 65)			Females (*n* = 230)		Total (*n* = 295)			
Genotype/Allele *	IFN-β R (*n* = 40)	IFN-β NR (*n* = 25)	*p*	IFN-β R (*n* = 133)	IFN-β NR (*n* = 97)	*p*	IFN-β R (*n* = 173)	IFN-β NR (*n* = 122)	*p*
CCR5 Δ32									
Codominant model									
wtwt	35 (87.5)	23 (92.0)	0.488	115 (86.5)	87 (89.7)	0.580	150 (86.7)	110 (90.2)	0.517
wtΔ32	5 (12.5)	2 (8.0)		17 (12.8)	10 (10.3)		22 (12.7)	12 (9.8)	
Δ32Δ32	0	0		1 (0.7)	0		1 (0.6)	0 (0.0)	
Dominant model									
wtΔ32 + Δ32Δ32	5 (12.5)	2 (8.0)	0.448	18 (13.5)	10 (10.3)	0.460	23 (13.3)	12 (9.8)	0.366
Recessive model									
wtΔ32 + wtwt	40 (100.0)	25 (100.0)	-	132 (99.3)	97 (100.0)	0.578	172 (99.4)	122 (100.0)	0.586
Overdominant model									
wtwt + Δ32Δ32	35 (12.5)	23 (8.0)	0.448	116 (87.2)	87 (89.7)	0.359	151 (87.3)	110 (90.2)	0.284
wt	93.8	96.0	0.451	92.9	94.8	0.392	93.1	95.1	0.313
Δ32	6.2	4.0		7.1	5.2		6.9	4.9	
CTLA-4 +49 A/G									
Codominant model									
AA	16 (40.0)	12 (48.0)	0.655	56 (42.1)	28 (28.9)	0.114	72 (41.6)	40 (32.8)	0.273
AG	18 (45.0)	11 (44.0)		54 (40.6)	50 (51.5)		72 (41.6)	61 (50.0)	
GG	6 (15.0)	2 (8.0)		23 (17.3)	19 (19.6)		29 (16.8)	21 (17.2)	
Dominant model									
AG + GG	24 (60.0)	13 (52.0)	0.583	77 (57.9)	69 (71.1)	0.039	101 (58.4)	82 (67.2)	0.123
Recessive model									
AG + AA	34 (85.0)	23 (92.0)	0.336	110 (82.7)	78 (80.4)	0.656	144 (83.2)	101(82.8)	0.920
Overdominant model									
AA + GG	22 (55.0)	14 (56.0)	0.937	79 (59.4)	47 (48.5)	0.100	101 (58.4)	61 (50.0)	0.154
A	62.5	68.0	0.382	62.2	54.9	0.094	62.2	57.8	0.256
G	37.8	32.0		37.8	45.1		37.8	42.2	
CCR5 Δ32/CTLA4 +49 A/G									
wtwt/AA	14 (35.0)	12 (48.0)	0.546	49 (36.8)	27 (27.8)	0.118	63 (36.4)	39 (32.0)	0.146
wtΔ32/AA	2 (5.0)	0 (0.0)		7 (5.3)	1 (1.0)		9 (5.2)	1 (0.8)	
wtwt/G+	21 (52.5)	11 (44.0)		66 (49.6)	60 (61.9)		87 (50.3)	71 (58.2)	
wtΔ32/G+	3 (7.5)	2 (8.0)		11 (8.3)	9 (9.3)		14 (8.1)	11 (9.0)	

* Data are presented as *n* (%). CCR5, chemokine receptor 5; CTLA-4, cytotoxic T-lymphocyte antigen-4.

## Data Availability

The data that support the findings of this study are available upon request from the corresponding author due to privacy and ethics concerns.
